# Prognostic value of the CRM-status in pancreatic ductal adenocarcinoma - data from a regional cancer registry

**DOI:** 10.1186/s12885-024-12995-z

**Published:** 2024-10-15

**Authors:** Jasmin Schuhbaur, Irina Surovtsova, Thomas Seufferlein, Daria Kokh, Gertrud Szotyori-Artz, Claudia Winzler, Juliane Schütz, Waldemar Uhl, Andrea Tannapfel, Philipp Morakis

**Affiliations:** 1 Klinik für Innere Medizin I, Universitätsklinikum Ulm, Albert-Einstein-Allee 23, D-89081 Ulm, Germany; 2 Klinische Landesregisterstelle Baden-Württemberg GmbH, Krebsregister Baden-Württemberg, Stuttgart, Germany; 3 Geschäftsstelle Qualitätskonferenzen bei der Klinischen Landesregisterstelle Baden-Württemberg GmbH, Krebsregister Baden-Württemberg, Stuttgart, Germany; 4 Klinik für Allgemein- und Viszeralchirurgie, Ruhr Universität Bochum, Standort St. Josef Hospital, Gudrunstraße 56, 44791 Bochum, Germany; 5 Institut für Pathologie der Ruhr-Universität Bochum am Berufsgenossenschaftlichen Universitätsklinikum, Bergmannsheil Bürkle-de-la-Camp-Platz 1, D-44789 Bochum, Germany

**Keywords:** Pancreatic cancer, Resection margin, R0 resection, Prognostic factors, CRM

## Abstract

**Background:**

Ductal pancreatic adenocarcinoma (PDAC) still has a dismal prognosis even when deemed resectable. A cancer free resection margin (R0) is associated with a more favourable prognosis than the presence of tumour cells at resection margin (R1). However, the precise definition of the R0 status is still a matter of debate in PDAC. For a more accurate determination of R0 in PDAC the concept of circumferential resection margins (CRM) has been established and has been incorporated into the German national S3 guideline on exocrine pancreatic cancer. However, an international standardized nomenclature of CRM is still missing, and the clinical value of the CRM concept is not yet fully established. Here we evaluate whether the CRM status as defined in the national German S3 guideline corresponds with overall and progression free survival in PDAC using data from the regional cancer registry of the State of Baden Württemberg in Germany.

**Methods:**

Data from the cancer registry of the State of Baden-Württemberg, Germany, were used to assess the relationship between CRM-status and progression free survival (PFS) as well as 3-year overall survival (OS) using documented patients diagnosed with resectable ductal adenocarcinoma of the pancreas between 2015 and 2020. Patients were residents of the State of Baden-Württemberg and underwent surgery for PDAC. The R-status was assessed according to the national German S3 guideline with R0 wide/CRM- when CRM is > 1 mm from the tumour, R0 narrow/CRM + when CRM is ≤ 1 mm from the tumour and R1 when tumour cells are found at the resection margin.

**Results:**

In total we identified 1098 cases surgically treated for pancreatic cancer and fulfilling the inclusion criteria. 340 patients had an R0 wide/CRM- resection, 410 patients an R0 narrow/CRM + resection, and 348 patients an R1 resection. The R0 wide/CRM- status was associated with a significantly increased median OS rate compared to the other two groups (51,5%, 37,4% and 26,7% for R0 wide/CRM-, R0 narrow/CRM + and R1, respectively). mPFS was also longer in the R0 wide/CRM- group. These findings were robust with regards to grading and tumour location.

**Conclusions:**

CRM is prognostic for patients with resectable PDAC making the pathological assessment of the R-status according to the CRM concept worthwhile.

## Background

Pancreatic ductal adenocarcinoma (PDAC) is the most common malignancy of the pancreas and still associated with a poor prognosis. The 5-year overall survival rate is approximately 10% [1, 2]. Surgical resection is the only potentially curative treatment for PDAC [1, 3] and attaining tumour free margins (R0) is recognized as an important factor for patient survival [4–6]. PDAC shows an infiltrative, discontinuous growth pattern with a marked desmoplastic stromal response making the evaluation of the CRM-status more difficult, but also relevant to properly define the resection margin [7]. Retrospective analyses suggest that not only a free resection margin, but also the presence of tumour cells in close vicinity of the resection margin has prognostic value [8]. Besides, different terminologies for microscopic margin involvement have emerged in the literature [9]. In 2006 Verbeke and colleagues proposed a standardized protocol for the examination of a pancreaticoduodenectomy specimen and redefined R1 as tumour cells within 1 mm of the resection margin and R0 as tumour cells > 1 mm distant from the resection margin [10]. Using this definition, they reported a trend towards a better median and overall survival after R0 resection. In 2014 the International Study Group of Pancreatic Surgery endorsed the definition of R1 resection (tumour at or < 1 mm from the margin) used by the British Royal College of Pathologists [11]. The UICC classifies only tumour cells at the definitive resection margin as R1. The use of these divergent definitions side by side in different studies makes it difficult to directly compare their results and has led to the fact that reported R1 resection rates vary considerably between studies [12, 13]. To comply with the UICC classification, but also to appreciate the fact that there could be a difference in outcome depending on the presence of tumour cells ≤ 1 mm or > 1 mm from the resection margin, the German S3 guideline adopted the concept of the circumferential resection margin (CRM) also for pancreaticoduodenectomy specimen examination [1]. The CRM assessment comprises an analysis of the anterior, medial and posterior surface as well as the uncinate process margin [14]. Using the CRM concept margins are further classified into R1 and R0 where R1 indicates tumour cells at the definitive resection margin and R0 is split into two groups: “R0 wide/CRM-“describing tumour cells > 1 mm from the resection margin and “R0 narrow/CRM+” indicating tumour cells ≤ 1 mm from the resection margin. While the CRM concept has been accepted for the pathological examination of a pancreaticoduodenectomy specimen, the prognostic value of the “1 mm concept” has not been unanimously confirmed [15, 16]. A recent systematic review showed that an R0 (CRM-) resection was independently associated with improved OS compared to R1 and R0 (CRM+), respectively [17]. Furthermore, the prognostic significance of CRM status in distal pancreatic ductal adenocarcinoma (PDAC) originating from the pancreatic tail/body remains controversial [18, 19]. We reasoned that data from a cancer registry might be informative to clarify the prognostic value of the R0 wide/R0 narrow concept in the pathological assessment of PDAC. To this end, we analysed the data of all patients with resectable ductal pancreatic adenocarcinoma documented in the cancer registry of the State of Baden Württemberg between 2015 and 2020.

## Methods

### Eligibility criteria and study population

Data were obtained from the cancer registry of the State of Baden Württemberg, Germany covering PDAC diagnoses from 2015 to 2020. Cases included in the analysis were from patients aged > 17 years and residing in the State of Baden Württemberg who had a histologically proven, resectable adenocarcinoma of the pancreas, pancreatic tumour resection as well as a complete pathology report including the unambiguous definition of R-status and CRM-status according to the German S3 guideline. Exclusion criteria were metastatic disease, neoadjuvant therapy or death within 30 days after diagnosis. Since the cancer registry database includes also patient records with missing data (e.g. the exact resection date), we employed the diagnosis date to exclude cases with reported death within the first weeks from the surgery. All data used in this study come from the cancer registry of the State of Baden Württemberg, Germany. According to the Baden-Württemberg State Cancer Registry Act, the physician or dentist must inform the patient of the intended or completed notification at the earliest possible time. As a rule, the notification must be made prior to the registration. The notification must state whether the patient has been informed of the notification. The patient may object in writing to the further processing of his/her identity data by the trust center, the clinical state registry and the epidemiological cancer registry to the doctor or dentist. The data was compiled by the Baden-Württemberg Cancer Registry itself. No other data was used for the analysis. Identity data are not available to the Clinical State Registry and were therefore not used. The data were only pseudonymized and used in aggregated form. Therefore, no ethical review and approval had to be obtained for this study. The present study complies with the relevant guidelines of the Declaration of Helsinki.

### Definition of CRM-status

The CRM-status was defined as stipulated in the German S3-guideline on exocrine pancreatic carcinoma in 2013: R0 wide/CRM-: tumour cells > 1 mm from the resection margin, R0 narrow/CRM +: tumour cells ≤ 1 mm from the definitive resection margin, R1: tumour cells at the definitive resection margin [20]. The CRM-status was extracted from full-text pathology reports using an automated text analysis program and manually annotated.

### End points

We assessed progression free survival (PFS), the 3-year overall survival (OS) rate as well as median OS of patients after R1, R0 narrow/CRM + or R0 wide/CRM- resection of PDAC. Furthermore, correlation to histological grading was performed. For definition of OS and PFS the time from diagnosis to an event was used. Events were defined as either local recurrence, distant recurrence, or reported death for PFS. For OS analysis reported death was considered as event.

### Statistical analysis

The Kaplan-Meier method and long-rank test were used to compare survival curves between the different resection states (R1, R0 narrow/CRM+, R0 wide/CRM-). A Cox proportional-hazards model was used to evaluate the effects of major clinical prognostic factors. A *p* value of less than 0.05 was considered to indicate statistical significance.

All statistical analyses were performed using the “survival” and “survminer” packages of R software version 4.1.1 (https://www.r-project.org/).

## Results

**Fig. 1:**
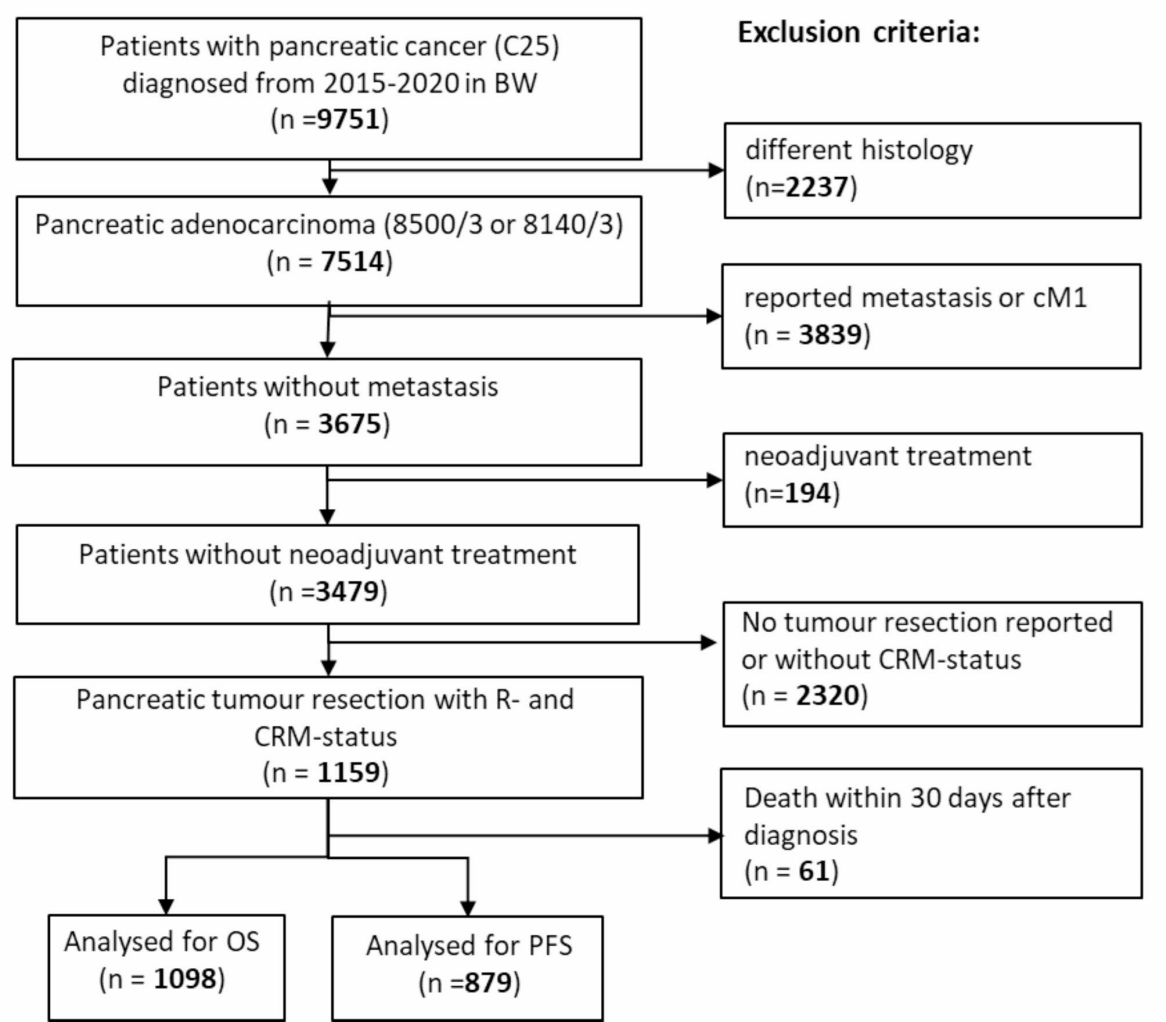
Flow chart of the patient selection. It is noteworthy, that since PFS analysis requires a complete follow-up in addition to the death record, the size of the PFS group is smaller than OS one

A total of 1098 cases registered between 2015 and 2020 and fulfilling the inclusion criteria could be included in the analysis (Fig. 1).

### Patient demographic and clinical information

Table 1 illustrates patient demographics and clinical information. Among the 1098 patients analysed, median age was 71 years, with a male to female ratio of 49–51%. Most tumours were located in the head of the pancreas (81%), 6% in the body and 7% in the tail, respectively. 6% had other or not specified locations. Data on TNM classification, grading and the resection status are shown in Table 1.

For tumours diagnosed in 2015 or 2016, the T category is classified according to the 7th edition of the TNM classification [21]. For tumours diagnosed starting from 2017, the T-category is classified according to the 8th edition [22].

The N category is always classified following the 8th edition of the TNM classification (pN1, metastasis in 1–3 LNs; pN2, metastasis in 4 or more LNs). There were more pT1 tumours and more patients with an N0 resection in the R0 wide group. Vice versa, the R1 group comprised more patients with pT4 and N2 tumours. Adjuvant chemotherapy was reported for 508 patients (46%). Gemcitabine-based regimens were reported for 71.6% and fluorouracil-based regimens for 28.3% of the patients. Fluorouracil-based therapy was mostly employed after 2018.

**Table 1:** Baseline clinical and patient characteristics

	All patients	R0 wide	R0 narrow	R1
Patients number (%)	1098(100%)	340(31%)	410(37%)	348(32%)
Patient age, median (SD)	71(10.1)	72(9.9)	70(10.5)	72(9.8)
Patient sex				
- Male (%)	542(49.4%)	165(48.5%)	207(50.5%)	170(48.9%)
- Female (%)	556(50.6%)	175(51.5%)	203(49.5%)	178(51.1%)
Tumour location				
- head (%)	888(80.9%)	278(81.8%)	326(79.5%)	284(81.6%)
- body (%)	66(6%)	14(4.1%)	25(6.1%)	27(7.8%)
- tail (%)	77(7%)	29(8.5%)	30(7.3%)	18(5.2)
- overlapping (%)	12(1.1%)	1(0.3%)	7(1.7%)	4(1.2%)
- undefined (C25.9) (%)	55(5%)	18(5.3%)	22(5.4%)	15(4.3%)
Tumour grade				
- 1 or 2 (%)	625(56.9%)	227(66.8%)	226(55.1%)	172(49.4%)
- 3 or 4 (%)	473(43.1%)	113(33.2%)	184(44.9%)	176(56.6%)
pN				
- pN0 (%)	287(26.1%)	132(38.8%)	97(23.6%)	58(16.7%)
- pN1 (%)	456(41.5%)	134(39.4%)	175(42.7%)	147(42.2%)
- pN2 (%)	355(32.3%)	74(21.8%)	138(33.7%)	143(41.1%)
pT (8th )				
- pT1	74(9.6%)	47(18.7%)	19(6.6%)	8(3.4%)
- pT2	439(56.9%)	148(59%)	178(62%)	113(48.3%)
- pT3	246(31.9%)	55(21.9%)	89(31%)	102(43.6%)
- pT4	12(1.6%)	1(0.4%)	0	11(4.7%)
pT (7th )				
- pT1	4(1.2%)	2(2.3%)	2(1.6%)	0
- pT2	8(2.5%)	5(5.6%)	1(0.8%)	2(1.8%)
- pT3	308(94.2%)	81(91%)	120(96.8%)	107(93.9%)
- pT4	7(2.1%)	1(1.1%)	1(0.8%)	5(4.4%)
Adjuvant therapy				
- Fluorouracil-based	144(28.3%)	48(31.4%)	59(28.8%)	37(24.7%)
- Gemcitabine-based	364(71.6%)	105(68.6%)	146(71.2%)	113(75.3%)
- Missing	590	187	205	198

### Relationship between CRM-status and overall survival

**Fig. 2:**
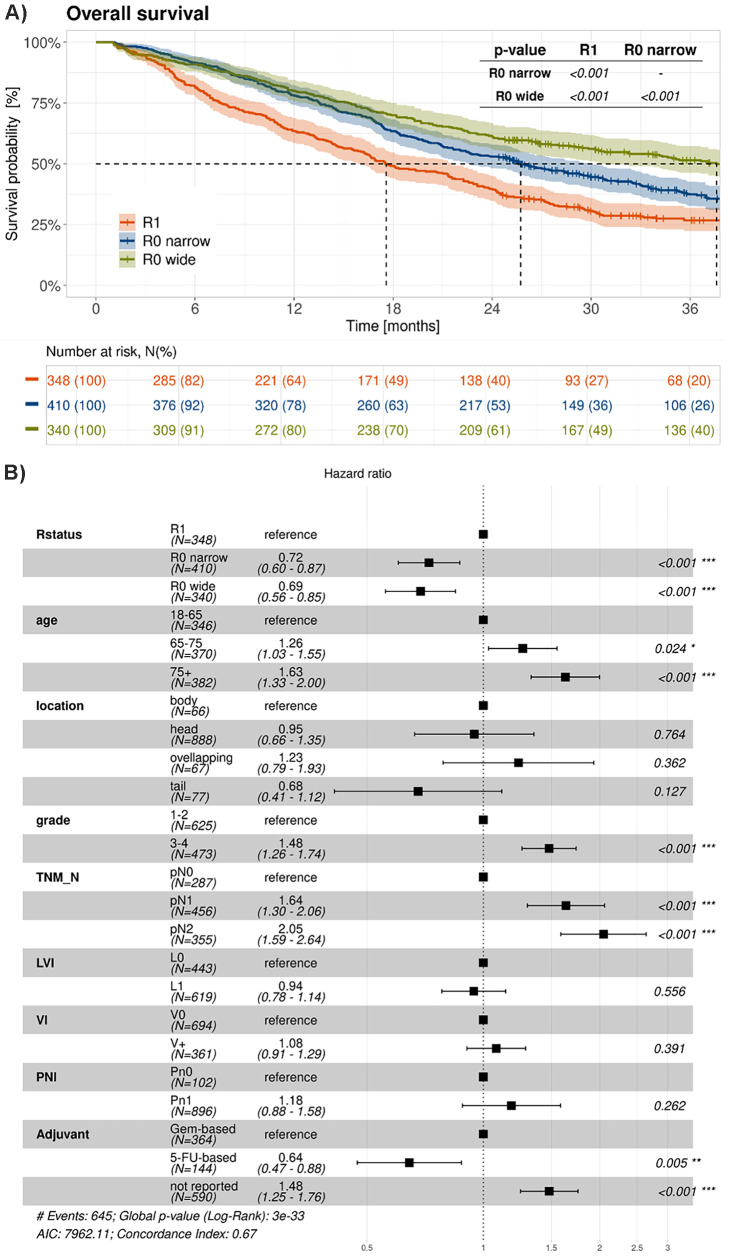
(**A**): 3-year-OS in R0/CRM-, R0/CRM + and R1 resected patients. Dotted lines refer to mOS for each curve. (**B**): Multivariate Cox regression analysis showed that apart from the R status, age, N-status, grading as well as adjuvant chemotherapy were important prognostic parameters. LVI stands for lymphovascular invasion, VI for venous invasion, and PNI for perineural invasion. 5-FU- and Gem-based refer 5-Fluorouracil- and Gemcitabine -based therapies, respectively

In the whole cohort, the 3-year OS rate was significantly different between the groups: 51.5% for R0 wide/CRM- (95% CI 46.3–57.2%), 37.4% for R0 narrow/CRM+ (95% CI 32.8–72.7%) and 26.7% for R1 (95% CI 22.3–32%), respectively (Fig. 2A). Using R1 as reference, the HR for R0 wide/CRM- was 0.66 (95% CI 0.55–0.81) and 0.77 (95% CI 0.65–0.92) for R0 narrow/CRM+, respectively. A COX regression analysis (Fig. 2B) showed that apart from the R status, N-status, grading as well as adjuvant chemotherapy were important prognostic parameters.

Median OS was 37.6 months (95% CI, 30.8 to 45.8) in the R0 wide/CRM- group, 25.7 months (95% CI, 22.6 to 29.9) in the R0 narrow/CRM + group and 17.6 months (95% CI, 16.1 to 21.6) in the R1 resected group, respectively. This difference in median OS between the three groups was statistically significant (*p* < 0.001 for R0 wide/CRM- and R0 narrow/CRM + and *p* < 0.001 for R0 narrow/CRM + and R1). The results of mOS in the different subgroups are summarized in the Table 2.

Systemic adjuvant therapy modalities were equally distributed between the CRM groups.

### Role of the tumour grading

**Fig. 3:**
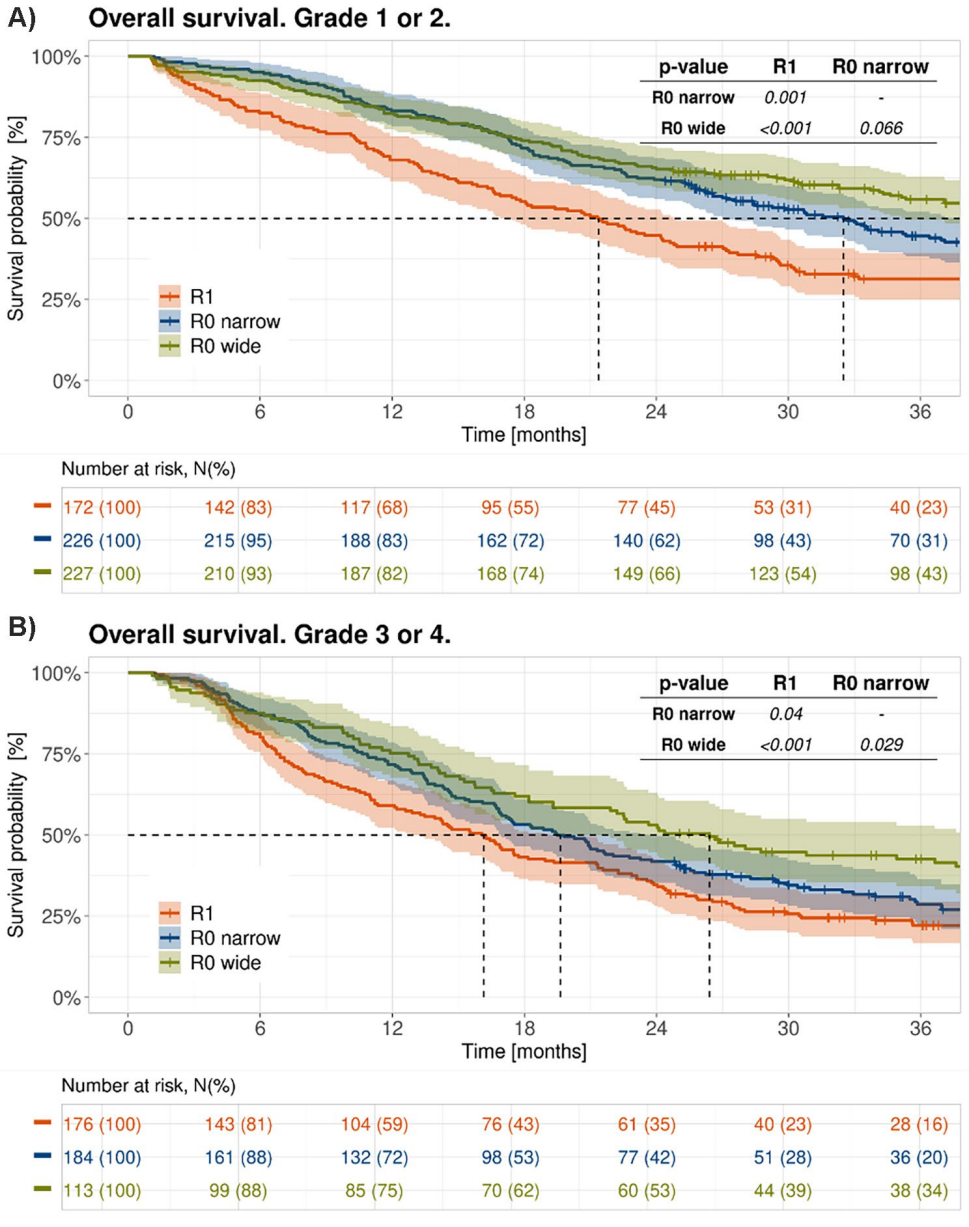
Overall survival of patients with an R0 wide/CRM-, R0 narrow/CRM + and R1 resection according to tumour grading. Dotted lines refer to mOS for each curve. **A**: Grade 1/2. **B**: Grade 3/4

The difference in mOS between the R0 CRM+/- and R1 groups was observed independently of tumour grading (grade 1/2: R0 wide/CRM- vs. R0 narrow/CRM + *p* = 0.0662; R0 narrow/CRM + vs. R1: *p* = 0.001; grade 3/4 tumours: R0 wide/CRM- vs. R0 narrow/CRM+: *p* = 0.029; R0 narrow/CRM + vs. R1: *p* = 0.04 (Fig. 2)). As expected, patients with grade 1/2 pancreatic cancers exhibited a longer mOS than those with grade 3/4 tumours. Median OS for grade 1/2 was 42.8 months (95% CI, 35.3 to 58.8) in the R0 wide/CRM- group, 32.5 months (95% CI, 27.2 to 39.5) in the R0 narrow/CRM + group and 21.4 months (95% CI, 17.4 to 24.8) in the R1-group (Fig. 3A). The respective mOS for grade 3/4 PDAC were 26.4 months (95% CI, 19.3 to 38.2) in the R0 wide/CRM- group, 19.6 months (95% CI, 16.9 to 23.8) in the R0 narrow/CRM + group and 16.1 months (95% CI, 12.9 to 19.3) in the R1 group (Fig. 3B). The difference in the 3-year OS rate between R0 wide/CRM- and R0 narrow/CRM + was more pronounced when tumours had a lower grading (G1/2 compared to G3/4; Fig. 2A and B; Table 2).

### Role of tumour location

**Fig. 4:**
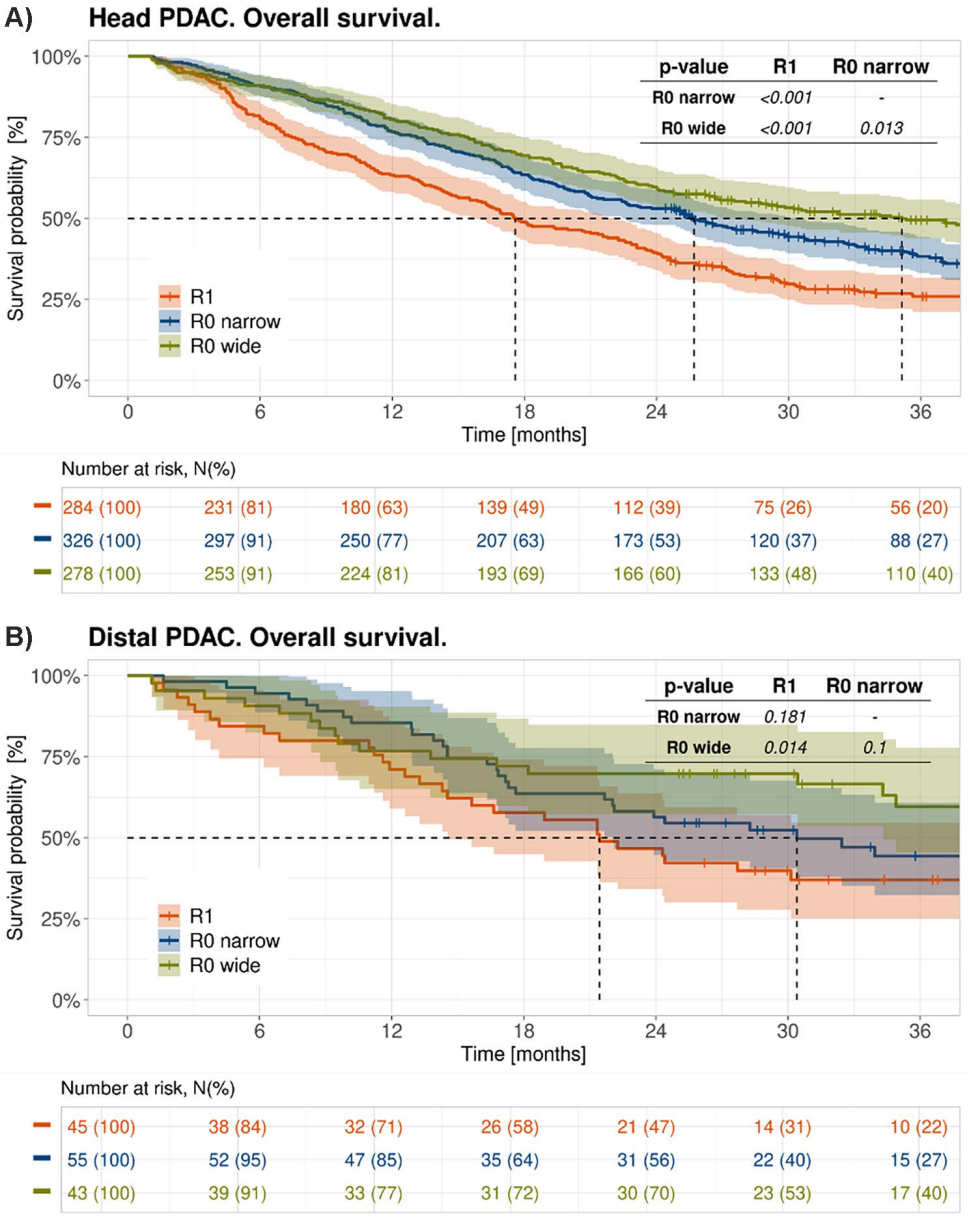
Overall survival of patients with an R0 wide/CRM-, R0 narrow/CRM + and R1 resection for different tumour locations. Dotted lines refer to mOS on each curve. (**A**): pancreatic head (**B**): tail/body

Another important aspect is the dependence of resection status and overall survival on tumour location. A statistically significant difference in mOS between the R0 CRM+/- and R1 groups was observed in pancreatic head tumours (R0 wide/CRM- vs. R0 narrow/CRM + *p* = 0.013; R0 wide/CRM + vs. R1 and R0 narrow/CRM + both *p* < 0.001 (Table 2)). The median OS for this subgroup was 35.1 months (95% CI, 27.5 to 45.2) in the R0 wide/CRM- group, 25.7 months (95% CI, 22.6 to 30.7) in the R0 narrow/CRM + group, and 17.6 months (95% CI, 15.9 to 22.3) in the R1 group (Fig. 4A).

In tumours of the tail/body, the three-year overall survival (OS) in margin-negative patients (R0 CRM-) was significantly longer compared to margin-positive patients (R1/R0 CRM+) (59.6% vs. 41%, *p* = 0.01). The longest median overall survival (mOS) was observed in the R0 wide/CRM- group, where mOS was not reached. This was followed by the R0 narrow/CRM + group with a mOS of 30.4 months (95% CI, 22.0 to 60.9), and the shortest mOS of 21.4 months was seen in the R1 resection group (95% CI, 14.5 to 42.3). However, likely due to the small sample size, this difference was not statistically significant (Fig. 4B).

**Table 2:** Median OS and mPFS according to the R status, location and tumour grading. “UCI” and “LCI” refer to the upper and lower confidence intervals

	*R* status	*N*	Median (months)	0.95 LCI	0.95 UCI
OS, any grading, any location	R0 wide	340	37.6	30.8	45.8
	R0 narrow	410	25.7	22.6	29.9
	R1	348	17.6	16.1	21.6
OS, Grade 1 or 2	R0 wide	227	42.8	35.3	58.8
	R0 narrow	226	32.5	27.2	39.5
	R1	171	21.4	17.4	24.8
OS, Grade 3 or 4	R0 wide	113	26.4	19.3	38.2
	R0 narrow	184	19.6	16.9	23.8
	R1	176	16.1	12.9	19.3
OS,	R0 wide	278	35.1	27.5	45.2
pancreatic	R0 narrow	326	25.7	22.6	30.7
head	R1	284	17.6	15.9	22.3
OS,	R0 wide	43	not reached	34.3	-
tail/body	R0 narrow	55	30.4	22.0	60.9
	R1	45	21.4	14.5	42.3
PFS, any grading, any location	R0 wide	288	32.3	24.4	44.3
	R0 narrow	328	19.1	16.3	22.8
	R1	263	14.1	12.2	16.3

### Relationship between CRM-status and progression free survival (PFS)

**Fig. 5:**
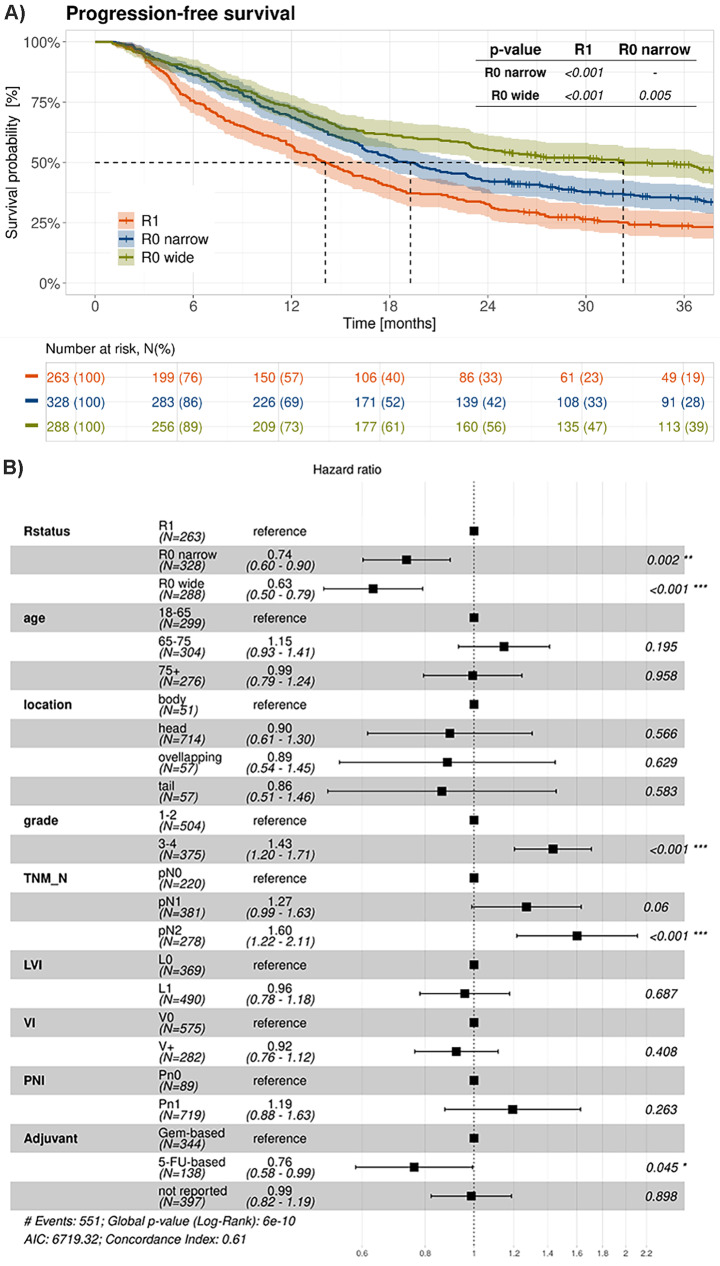
PFS in R0/CRM-, R0/CRM + and R1 resected patients: (**A**) Kaplan-Meier survival curves and (**B**) Multivariate Cox regression analysis. LVI stands for lymphovascular invasion, VI for venous invasion, and PNI for perineural invasion. 5-FU- and Gem-based refer 5-Fluorouracil- and Gemcitabine -based therapies, respectively

PFS was also statistically significantly longer in R0 wide/CRM- resected patients compared to both R0 narrow/CRM + and R1 resected patients, respectively (Fig. 5A). mPFS was 32.3 months (95% CI, 24.4 to 44.3), 19.1 months (95% CI, 16.3 to 22.8), and 14.1 months (95% CI, 12.2 to 16.3) in the respective groups (*p*-value < 0.001 in both cases; Table 2). The corresponding 3-year PFS rates were 49%, 35% and 23% for R0 wide/CRM-, R0 narrow/CRM + and R1 resected patients, respectively. A COX regression analysis (Fig. 5B) showed that apart from the R status, N-status, grading as well as adjuvant chemotherapy were important prognostic parameters also for PFS.

## Discussion

PDAC has still a dismal prognosis even if resectable. R0 resection is an important goal in PDAC surgery. To refine the assessment of the R status after PDAC surgery the CRM concept was introduced and recommended by the German S3 guideline in 2013. Here we analysed data of over 1000 cases from a regional cancer registry to assess the prognostic value of the CRM concept in the pathological assessment of PDAC and thereby verify this recommendation. The univariate analysis of the data shows that both, PFS and OS, significantly correlate with the state of the refined resection status, R0 wide/CRM-, R0 narrow/CRM + and R1, respectively, indicating that the precise assessment of the resection margin has indeed prognostic value. The R0 wide/CRM- status was associated with the longest PFS and OS whereas an R1 resection had the poorest outcome. The outcome of patients with R0 narrow/CRM + resection status was in between the two other groups. These results could be confirmed also when tumour grading was additionally considered. Our results are in line with the increasing evidence that the presence of tumour cells in the close vicinity of the resection margin has prognostic value [23] reflecting the invasive nature of PDAC. Our analysis also shows that the definition of the R status used in the national German S3 guideline properly discriminates the different states at the resection margin and that the workup of the pathological specimen as recommended by the guideline is useful to predict patient prognosis. Of note, there are still subtle differences regarding the definition of the R-status in the literature since some definitions count a distance of exactly 1 mm as “R0 wide”, whereas the definition used by the German S3 guideline defines “R0 wide” as tumour cells > 1 mm from the circumferential resection margin [1].

The data presented here clearly support the development of novel approaches aiming at increasing the rate of R0 wide/CRM- resections, improving local control and ideally overall survival in PDAC. One approach is the so called mesopancreatic excision that has a benefit especially for local control of posterior and medial resection margins, respectively [17, 24]. Another strategy is neoadjuvant chemotherapy or radiochemotherapy both of which have demonstrated to increase R0 resection rates in PDAC compared to immediate upfront surgery [25, 26].

The present study was performed using real world data reflecting actual clinical settings. The results obtained are in good agreement with data from clinical trials, including the prognostic role of the T- and N- Status as well as the efficacy of adjuvant chemotherapy protocols used. 5-FU based adjuvant treatment was mainly mFOLFIRINOX after publication of the PRODIGE24 trial and did show better outcome as compared to gemcitabine-based treatments. In conclusion, our data also demonstrate that clinical cancer registries provide a valuable source of information also when clinical trials are lacking or limited.

## Data Availability

No datasets were generated or analysed during the current study.
